# The primary cilium is a self-adaptable, integrating nexus for mechanical stimuli and cellular signaling

**DOI:** 10.1242/bio.014787

**Published:** 2015-11-24

**Authors:** An M. Nguyen, Y.-N. Young, Christopher R. Jacobs

**Affiliations:** 1 Department of Biomedical Engineering, Columbia University, New York, NY 10027, USA; 2 Runway Program, Jacobs Technion-Cornell Innovation Institute, Cornell Tech, New York, NY, 10011USA; 3 Department of Mathematical Sciences, New Jersey Institute of Technology, Newark, NJ, 07102USA

**Keywords:** Acetylation, Adaptation, Mechanosensing, Primary cilia

## Abstract

Mechanosensation is crucial for cells to sense and respond to mechanical signals within their local environment. While adaptation allows a sensor to be conditioned by stimuli within the environment and enables its operation in a wide range of stimuli intensities, the mechanisms behind adaptation remain controversial in even the most extensively studied mechanosensor, bacterial mechanosensitive channels. Primary cilia are ubiquitous sensory organelles. They have emerged as mechanosensors across diverse tissues, including kidney, liver and the embryonic node, and deflect with mechanical stimuli. Here, we show that both mechanical and chemical stimuli can alter cilium stiffness. We found that exposure to flow stiffens the cilium, which deflects less in response to subsequent exposures to flow. We also found that through a process involving acetylation, the cell can biochemically regulate cilium stiffness. Finally, we show that this altered stiffness directly affects the responsiveness of the cell to mechanical signals. These results demonstrate a potential mechanism through which the cell can regulate its mechanosensing apparatus.

## Abstract (precis)

**Summary:** Primary cilia stiffness can be modulated, affecting the cellular responsiveness to mechanical signals. We identify a potential mechanism that regulates cellular mechanosensitivity.

## INTRODUCTION

Cellular sensing of physical cues is essential to homeostasis and its dysfunction leads to devastating diseases, including atherosclerosis, osteoporosis, and cancer (Hoffman et al., 2011). Many cells are continuously challenged by stimuli, for example the disturbed blood flow with atherosclerosis, the disuse and resorption of bone tissue with osteoporosis, and the tissue stiffening with tumor progression. It is crucial that these cells respond and adapt to maintain homeostasis. In this context, we define sensory adaptation to be sensitivity adjustments to environmental conditions (Kurahashi and Menini, 1997). This type of adaptation enables cellular sensors to function within a wide range of stimuli intensities. For example, the opening and closing of large and small conductance mechanosensitive channels allow bacterial cells to maintain consistent internal pressure even in extreme environments. But under constant external pressure, these channels can desensitize or completely inactivate (Anishkin and Sukharev, 2009). Sensory adaptation in response to chemical and photostimuli has been well studied in specialized sensory cells, including those of auditory, olfactory and retinal systems (Condon and Weinberger, 1991; Kurahashi and Menini, 1997; Pugh et al., 1999). In olfactory sensory receptor cells, ligand-gated channels facilitate the chemosensing response to odorant stimuli and a single exposure can alter ligand affinity, reducing the odorant-induced action potential (Kurahashi and Menini, 1997). Even in the bacterial mechanosensitive channels, the most studied mechanosensor, it is remarkable the evidence for adaptation remains controversial and no clear mechanism has been identified to date (Naismith and Booth, 2012).

While ion channels are the best characterized and understood class of mechanosensors (Hirata et al., 2014), cells can detect physical cues with a diverse array of non-channel mechanosensors. A particularly interesting subset is structural mechanosensors that sense mechanical stimuli and also bear load. Examples of these structural mechanosensors include the focal adhesions that connect cells to extracellular matrix and the cytoskeleton that gives cells their structure. Adaptation in structural mechanosensors has previously been reported. For example, recruitment of vinculin can enlarge and thicken focal adhesions in response to mechanical stimuli (Galbraith et al., 2002). While this is suggestive that vinculin structurally reinforces adhesions, the mechanism responsible for this has not been identified and it remains unclear if this affects cellular responsiveness. Here, we show that the primary cilium is an adaptive mechanosensor and reveal a specific mechanism that can regulate cellular mechanosensitivity.

The primary cilium is a solitary, immotile multifunctional organelle that projects from nearly every cell in the human body during interphase (Kobayashi and Dynlacht, 2011). Not surprisingly, the cilium's dysfunction leads to a broad class of human diseases termed ciliopathies. As mechanosensors, primary cilia deflect in response to fluid flow, touch, vibration and pressure. In the kidney and liver, primary cilia transduce the flow rate of urine and bile (Masyuk et al., 2006; Praetorius and Spring, 2001), respectively. In the embryonic node, cilia detect the direction of nodal flow involved in tissue patterning (McGrath et al., 2003). Other studies have suggested the cilium's capacity to adapt and affect cell responsiveness. The cyclic AMP signaling response to primary cilium deflection (Besschetnova et al., 2010; Kwon et al., 2010) is also thought to regulate cilium length (Besschetnova et al., 2010; Ou et al., 2009) and longer cilia are more sensitive (Besschetnova et al., 2010; Rydholm et al., 2010). Together, these studies suggest length is one mechanism by which the cilium may adapt and regulate mechanosensitivity.

## RESULTS AND DISCUSSION

**Fig. 1.**
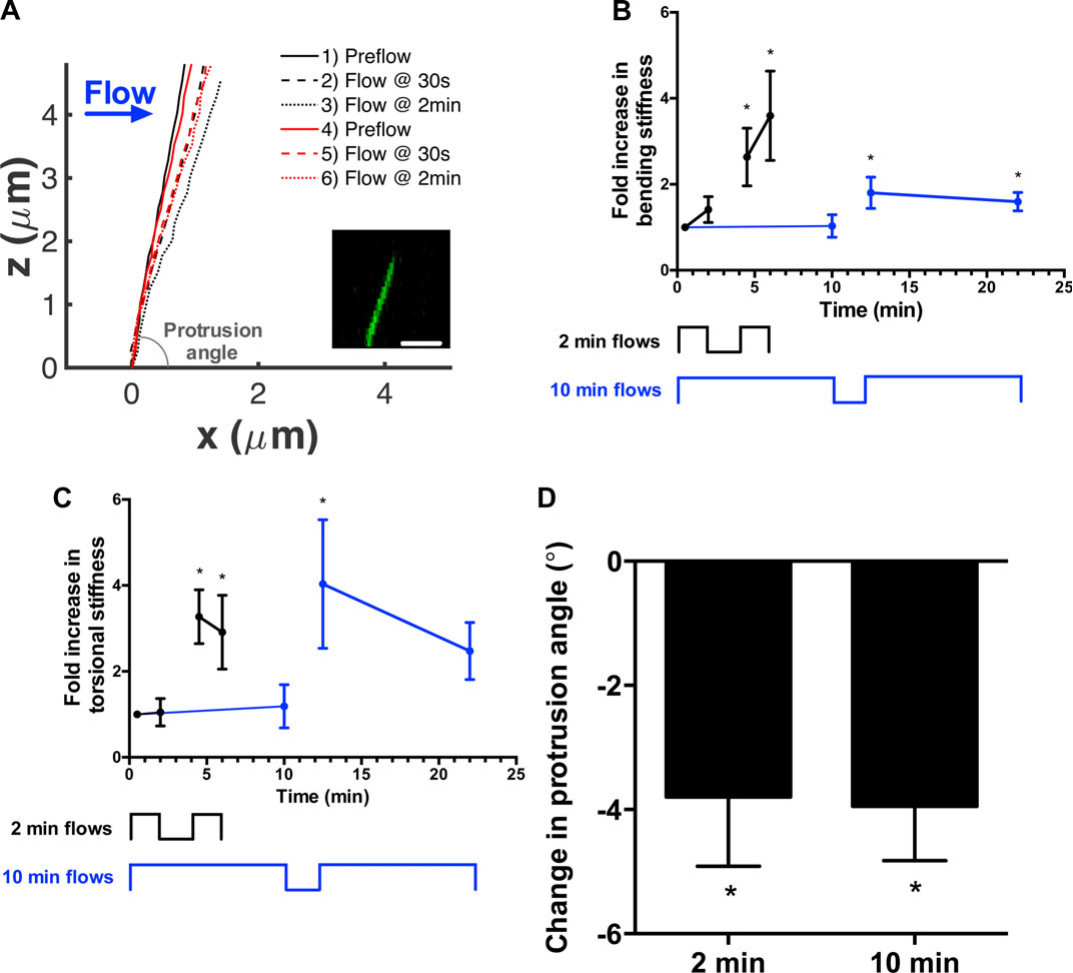
**Flow stiffens and rotates primary cilia.** (A) Representative profiles of a cilium through two bouts of 2-min flow. For each bout of flow, the cilium is graphed at rest (solid line), at 30 s of flow (dashed line), and 2 min of flow (dotted line). The first 2-min flow is in black while the second 2-min flow is in red. The inset is of a representative fluorescence micrograph from which cilium position is determined. The *x*-axis is positioned at the junction of the cilium and the cell and the protrusion angle measures the orientation of the cilium with respect to the cell. Scale bar: 2.5 µm. (B) Cilia were exposed to 2-min (black) or 10-min (blue) bouts of flow separated by 2 min of rest. The bending stiffness of the cilium shaft was measured and normalized to the first measurement at 30 s. *P<0.05, *n*=6 per group. Stiffness increased with exposure to flow, but the increase is independent of duration of flow exposure. (C) Similarly, torsional stiffness anchoring the cilium increased after each rest period independently of flow exposure. (D) Plastic deformation of the cilium was observed in the protrusion angle, the angle between the cilium and the cell membrane. The resting position of the cilium changes with exposure to flow, decreasing the protrusion angle. *P<0.05 from initial resting position of cilium, *n*=6 per group. Data presented as mean±s.e.m.

We recently observed that cilia deflected by fluid flow often did not recover to their original positions after flow had ceased (Downs et al., 2014), suggesting flow can induce ciliary structural reorganization. We hypothesized that this was a feature of the cilium's sensory adaptation and the cilium may alter its mechanical properties in response to stimuli. To test this, we first examined the biomechanics of the cilium by measuring changes in deflection with exposure to flow using a combined experimental and computational approach we previously developed (Downs et al., 2014; Young et al., 2012). Mouse inner medullary collecting duct (IMCD) cells transfected with a primary cilia live-cell marker somatostatin 3 receptor fused to GFP (SSTR3-GFP) were exposed to flow. We captured the cilium's 3D position at rest and under flow (Fig. 1A) to extract its mechanical properties by fitting its deflection to the predictions of a computational model, where in mechanical properties were systematically varied. Briefly, the model consisted of a cylindrical elastic beam anchored by a torsional spring (Young et al., 2012). We applied two bouts of flow and when comparing the deflections between bouts, we found stiffness along the ciliary shaft and at the basal anchorage increased, 2.6±0.7 and 3.3±0.6 times (*n*=6, Fig. 1B,C), respectively. This stiffening in response to deflection confirms the observations from our previous work (Downs et al., 2014). We then repeated the experiment with longer bouts of flow lasting 10 min and found cilia also stiffened, 1.8±0.4 times along the ciliary shaft and 4.0±1.5 times at the base (*n*=6, Fig. 1B,C). Thus, longer periods of flow did not further stiffen cilia, suggesting that the adaptation occurred quite rapidly. We also confirmed previous observations that flow altered the resting configuration (Downs et al., 2014). Specifically, after flow, the cilium's protrusion angle, the angle between the cilium and the cell, decreased 3.9±0.6° with no difference between 2 or 10-min bouts of flow (Fig. 1A,D). The decrease in protrusion angle was accompanied by a reduced angular deflection and an increase in torsional stiffness with the second bout of flow. Torque did not significantly change with flow. Recently, Battle et al. (2015) identified a pivot point for the cilium below the apical surface that can contribute to ciliary mechanics. We determined the pivot point for each bout of flow by calculating the instant center of rotation (Spiegelman and Woo, 1987). Though we observed the pivot point can fluctuate with flow, we did not find any statistically significant trends. Pooling the data, we found the pivot point for the cilium was 0.81±0.13 µm (*n*=12) below the cell membrane.

These data show the cilium can adapt to flow by altering its stiffness and orientation. Interestingly, these adaptations have occurred on a time scale of minutes. A previous report of flow-induced changes in cilium length occurred over a period of 3 h (Besschetnova et al., 2010). We did not observe changes in cilium length during our experiments, suggesting that ciliary adaptation can occur over a range of time scales, enabling this organelle to operate in a breadth of mechanoenvironments.

**Fig. 2.**
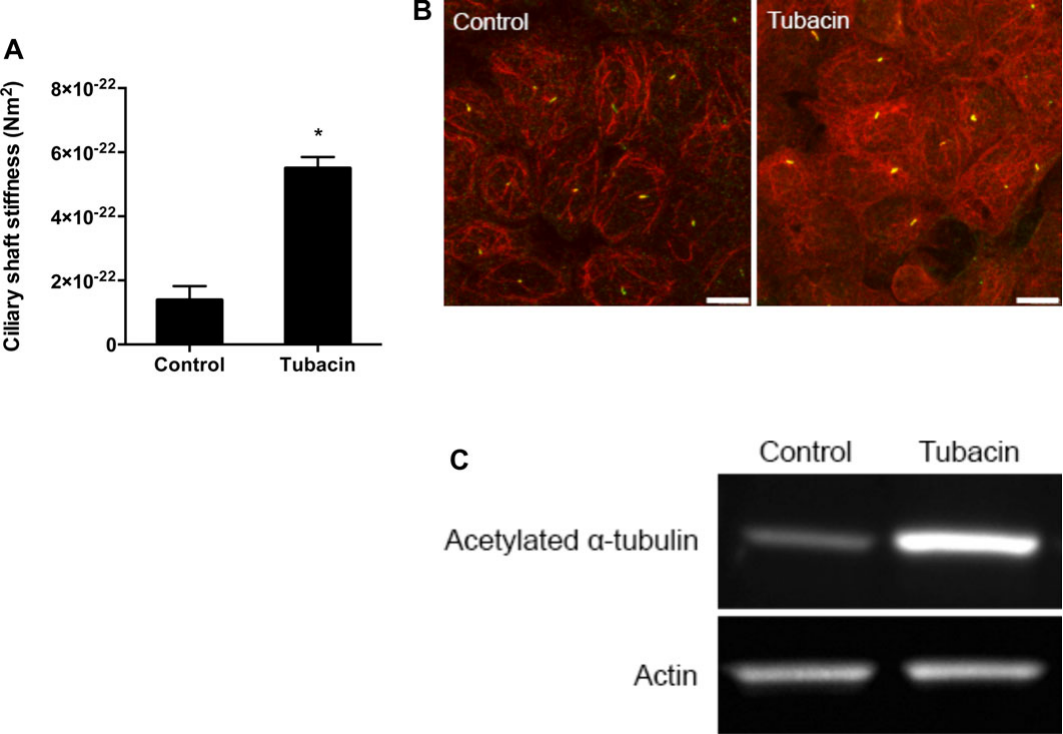
**Acetylation stiffens primary cilia.** (A) Ciliary bending stiffness calculated for the cells treated with niltubacin (control) and tubacin. Tubacin treatment increased rigidity by 4-fold. Data presented as mean±s.e.m.; *P<0.05, *n*=5 per group. (B) Immunostaining against acetylated α-tubulin (red) with cilia marked by SSTR3-GFP (green). The strong increase staining indicates increase in acetylation. Scale bar: 10 µm. (C) Protein expression of acetylated α-tubulin and actin was measured with western blot. Consistent actin bands show the same amount of protein was loaded while strong acetylated α-tubulin band with tubacin treatment confirms increased acetylation previously shown with immunocytochemistry.

In light of mechanical stimuli modulating stiffness, we suspected that structural changes driven by chemical stimuli could similarly stiffen the cilium. Nine microtubule doublets serve as the basis for the cilium's integrity (Schwartz et al., 1997) and acetylation and other post-translational modifications of tubulin subunits have been associated with increased microtubule stiffness (Felgner et al., 1996; Hawkins et al., 2013). Furthermore, mechanical stimuli can increase microtubule acetylation (Geiger et al., 2009; Li et al., 2011), suggesting adaptation of the cell to physical cues through an acetylation-mediated mechanism. Thus, we hypothesized that tubulin acetylation might also affect cilium stiffness. We treated cells with tubacin, a potent pharmacological deacetylation-inhibiting agent. Unlike other inhibitors that affect both tubulin and chromatin acetylation, tubacin specifically inhibits deacetylation of α-tubulin by binding to the α-tubulin catalytic domain of histone deacetylase 6 (HDAC6) (Haggarty et al., 2003). We treated IMCD cells with tubacin or niltubacin, an inactive analogue, and exposed them to flow. We observed a 4.0±1.3 fold increase in ciliary stiffness with tubacin compared with niltubacin (*n*=5 per group, Fig. 2A). The increase in acetylation was verified by immunocytochemistry and western blot (Fig. 2B,C). Here, we demonstrated that increasing acetylation of tubulin stiffens the cilium, suggesting one mechanism by which the cilium may alter its deflection.

**Fig. 3.**
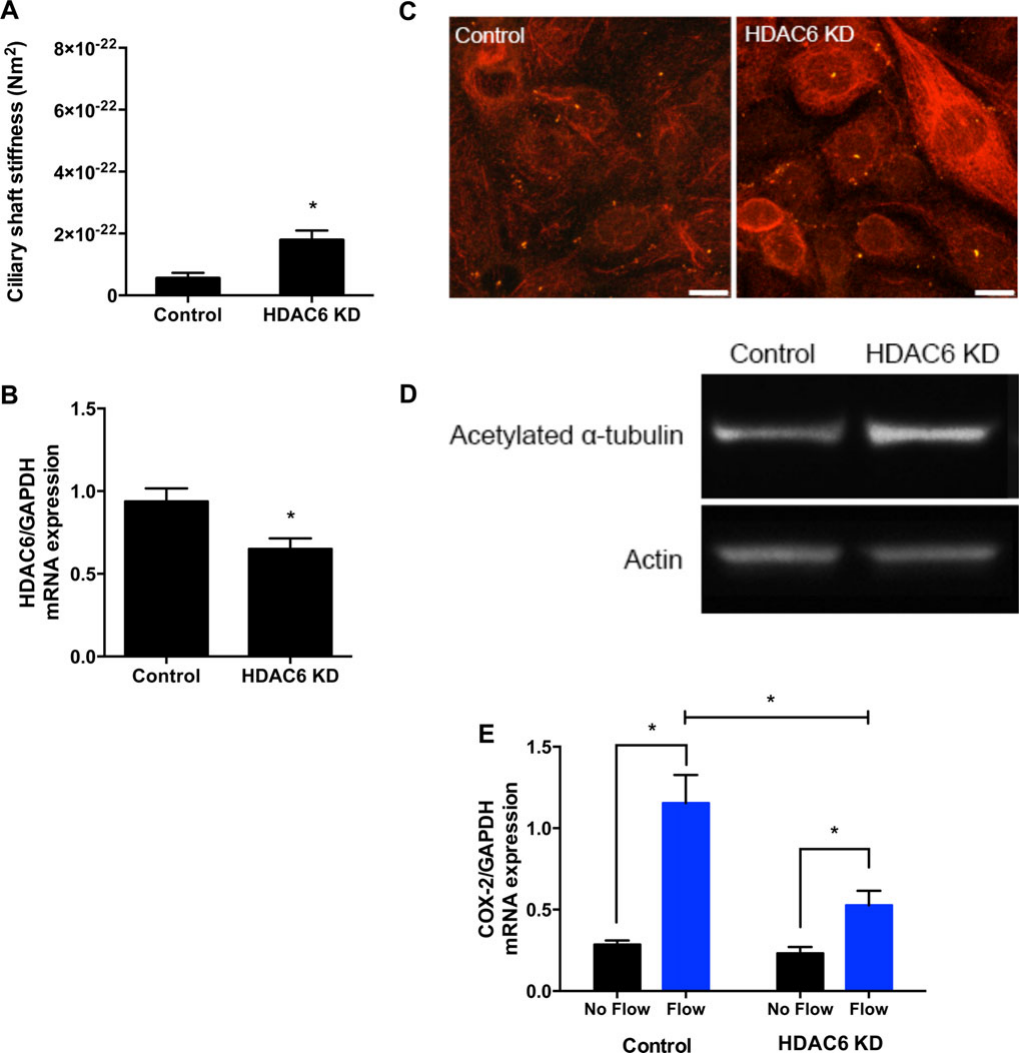
**The cell's internal mechanism to regulate acetylation can alter cilium stiffness and decrease mechanosensitivity.** (A) Cilium stiffness was measured in cells transfected with *HDAC6* siRNA and scrambled control. Knockdown of *HDAC6* resulted in a 3-fold increase in stiffness. (B) *HDAC6* mRNA expression normalized by housekeeping gene *GAPDH* was measured by qPCR in control and knockdown cells. Transfection resulted in a limited knockdown in *HDAC6* mRNA expression. (C) Immunocytochemistry with acetylated α-tubulin staining (red) and SSTR3-GFP cilia marker (green). Increased acetylated α-tubulin staining is observed in some cells. Scale bar: 10 µm. (D) Western blot probing for acetylated α-tubulin and actin. The stronger acetylated α-tubulin band in the knockdown cells confirms increased acetylation. (E) *COX-2* expression measured by qPCR with and without flow in cells transfected with *HDAC6* siRNA and scrambled control. A reduction of flow-induced increase in *COX-2* expression is indicative of reduced cell responsiveness with increased acetylation. Data presented as mean±s.e.m.; *P<0.05, *n*=5 per group.

We next hypothesized that the cell's internal mechanisms to regulate acetylation are sufficient for ciliary stiffening. We transfected IMCD cells with siRNA against *HDAC6*, which encodes a microtubule-associated deacetylase (Haggarty et al., 2003), or with a scrambled control, and found a 2.7±0.9 fold increase in stiffness (*n*=5 per group, Fig. 3A). We confirmed the knockdown with relative quantitative real-time RT PCR (qPCR) and found a 30.5±8.9% decrease in *HDAC6* expression (*n*=10 per group, normalized by housekeeping gene *GAPDH* expression, Fig. 3B). While not a complete knockdown, we still observed increased acetylation with immunocytochemistry and western blots (Fig. 3C,D) and not surprisingly, siRNA-mediated acetylation was more modest and less consistent when compared to tubacin-mediated acetylation. Nonetheless, these data show that the cell's endogenous regulation of acetylation can modulate cilium stiffness, advancing a specific mechanism of cilium-mediated adaptive cellular mechanosensing.

Next, we asked whether these primary cilium structural adaptations could decrease the responsiveness of the mechanosensing apparatus. We used *cyclooxygenase-2 *(*COX-2*)** expression, known to be regulated by flow (Flores et al., 2012), as an indicator of responsiveness to flow. COX-2 is an inducible enzyme that produces prostaglandins important in regulation of renal hemodynamics and inflammation, including in increasing renal blood flow and glomerular filtration rate (Harris, 2006). IMCD cells transfected with *HDAC6* siRNA or scrambled control were cultured and exposed to 1 h of oscillatory flow in parallel-plate flow chambers. Increased acetylation, as a result of *HDAC6* siRNA-mediated knockdown, inhibited flow-induced increases in *COX-2* expression by 55.9±16.3% (*n*=5 per group, normalized by housekeeping gene *GAPDH* expression; Fig. 3E). Inhibition of *HDAC6* can affect the glucocorticoid receptors and in turn, affect anti-inflammatory responses like *COX-2* expression (Kovacs et al., 2005; Zhang et al., 2008). However, other studies have shown that flow-induced *COX-2* expression is largely dependent on extracellular signal-regulated kinase and Protein kinase A pathways (Wadhwa et al., 2002a,b). Together these data demonstrate that increases in acetylation stiffen the cilium and lead to decreased cellular responsiveness to flow.

In light of our findings, we propose acetylation is a mechanism enabling the cilium to stiffen with mechanical stimuli and regulate cellular mechanosensitivity. Specifically, when perturbed with flow, primary cilia can increase acetylation and strengthen their microtubule-based structure. This reinforcement leads to decreased deflection to future mechanical stimuli, decreasing cellular sensitivity. While our data do not specifically connect acetylation with deflection, other groups have reported physical stimuli decreased HDAC6 activity and increased acetylation (Geiger et al., 2009; Li et al., 2011). Acetylation of tubulin has been implicated in microtubule stiffness (Felgner et al., 1996; Hawkins et al., 2013) and our results corroborate this. Although there is no direct connection between acetylation and mechanical properties (Howes et al., 2014), several potential mechanisms have been proposed. Acetylation occurs within the microtubule lumen at lysine-40 and, surprisingly, does not affect gross morphology or polymerization (Howes et al., 2014; Soppina et al., 2012). Acetylation may affect tubulin subunit interactions and access to the luminal surface for proteins. Recruitment of microtubule-associated proteins (MAPs) has been associated with acetylation and their binding to microtubules can increase microtubule stiffness nearly 4-fold (Felgner et al., 1997). In the future, the coupling of acetylation and mechanical properties at a molecular scale may best be shown with multiscale modeling. For example, coarse-grained simulations of tubulin dimers showed tubulin hydrolysis results in a bent conformation and simulations of molecules within each tubulin subunit revealed subunit-specific deformation patterns (Mitra and Sept, 2008).

Although our data suggest that axonemal tubulin acetylation is one mechanism by which cellular mechanosensitivity can be regulated, it is not our intention to attribute all primary cilium mechanics to this mechanism. For example, our data show that it did not affect basal mechanics such as torsional stiffness or changes in cilium orientation. Instead, these changes may be explained by a different mechanism. During ciliogenesis, the mother centriole nucleates the primary cilium and transforms into the basal body with numerous anchoring structures, including basal feet and striated rootlets, and nucleates the primary cilium (Kobayashi and Dynlacht, 2011). These anchors establish the positioning of the mother centriole and may also define the cilium orientation (Farnum and Wilsman, 2011). Cilium anchorage stiffness and orientation are believed to be regulated by changes in the number and distribution of appendages (Boisvieux-Ulrich et al., 1991; Kwon et al., 2011), which may also explain the flow-induced cilium rotation observed here. Although the connection between the basal body and the cell's microtubule network remains poorly understood, cytoskeletal microtubules are likely to form a crucial foundation upon which the cilium is constructed. The recent report on the impact of a sub-membrane pivot point on cilium stiffness supports this (Battle et al., 2015). Though we did not find a relationship between the position of the pivot point and duration of flow, it is possible that consistent changes require a longer stimulation duration. Additionally, the microtubule network extending from the basal body increases in density at the ciliary base with flow-induced rotation (Espinha et al., 2014). These increased attachments may stiffen the basal anchorage and lead to a decreased cellular response to mechanical stimuli. Interestingly, we observed different stiffening trends with the changes in duration of flow but did not find any statistically significant relationships. Similarly, the duration of flow affected the number attachments at the basal anchorage (Espinha et al., 2014). The sensitivity of the cilium to specific flow modalities remains unclear. Future studies investigating the effect of flow modality, including duration and intensity, can elucidate this.

Collectively, our data suggest a specific biomechanical adaptation model for sensory modulation of the primary cilium. We have shown that cilium stiffness changes in response to mechanical and chemical stimuli. We have also identified an acetylation-mediated mechanism through which the cell can regulate ciliary stiffness and in turn, regulate cellular responsiveness. Our experimental and computational techniques reveal sensory adaptation occurs on a surprisingly short time scale, making mechanosensors appealing therapeutic targets to the devastating disorders involving impaired cellular mechanosensitivity. Because we can already direct proteins to the organelle (Pazour and Bloodgood, 2008), the primary cilium is a particularly attractive target. Although attractive, mechanosensors are underrepresented as therapeutic targets and future work is needed to realize their full potential.

## MATERIALS AND METHODS

### Cell culture

Mouse inner medullary collecting duct (IMCD) cells transfected with somatostatin receptor 3 fused to GFP were a generous gift of Bradley K. Yoder of University of Alabama at Birmingham. Cells were cultured on fibronectin-coated coverslips and slides to 70% confluence in growth medium (DMEM F-12 with 10% FBS, 1% pen/strep and 200 µg/ml geneticin) and serum-starved for 72 h to promote cilia formation. For the tubacin experiment, cells were cultured as described above and treated with 0.5 mM of tubacin or niltubacin (Enzo Life Sciences) for 4 h prior to exposure to flow. For the *HDAC6* knockdown experiment, cells were cultured to 60% confluence in growth media and transfected with scrambled control or *HDAC6* siRNA (sc35545; Santa Cruz Biotechnology) using Lipofectamine 2000 (Life Technologies). Cells were serum-starved the following day for 72 h and then used in flow experiments. The average cilium length measured during the flow experiments was 3.9±0.2 µm (*n*=33).

### Fluid flow

To assess cilium deflection, steady fluid flow was applied to cells using a laminar flow chamber designed for confocal imaging (RC-30; Warner Instruments) and a syringe pump (GeniePlus; Kent Scientific) (Downs et al., 2014; Young et al., 2012). A 10 ml syringe (Norm-Ject; Air-Tite) was used to apply flow medium (DMEM F-12 without Phenol Red) at a rate of 0.5 ml/min, corresponding to 0.25 Pa of wall shear stress used in previous studies (Downs et al., 2014; Young et al., 2012). For the 2-min bouts of flow, flow was applied for 2 min, stopped for 2 min and applied for another 2 min. For the 10 min bouts, flow was applied for 10 min, stopped for 2 min and applied for an additional 10 min.

To quantify flow-induced gene expression, oscillatory fluid flow was applied to cells using large parallel plate flow chambers as previously described (Jacobs et al., 1998). Briefly, cells seeded on slides were placed in each flow chamber, incubated for 30 min and exposed to 1 h of oscillatory fluid flow at 1 Hz with a peak shear stress of 1 Pa. Flow parameters were chosen to correspond to a previous study finding an increase in microtubule density at the cilium base with flow (Espinha et al., 2014). Immediately after exposure to flow, slides were washed with PBS and cells were lysed for RNA extraction.

### Imaging and post-processing

A high-speed laser scanning confocal microscope with a 16 Hz bi-directional resonant scanner and a 100× oil objective (1.46 NA) was used to collect 3D images of primary cilia (512×512 *z*-stacks with a 0.17 µm slice thickness, TCS SP5; Leica Microsystems). Each *z*-stack was acquired in approximately 3 s. Cilia can be visualized with fluorescence microscopy due to the somatostatin receptor 3 GFP fusion protein targeted to the organelle (excitation: 488 nm, emission: 509 nm). Images were post-processed as previously described (Downs et al., 2014; Young et al., 2012). Briefly, a Gaussian filter was applied followed by a threshold. To determine the center of the cilium within each slice of the *z*-stack, the *x* and *y* coordinates of the pixels with an intensity value above the threshold were averaged.

### Deflection analysis

The model used to approximate cilium mechanics is described in detail in a previous paper, where the cilium is represented as a cylindrical elastic beam coupled to a rotational spring under hydrodynamic load (Young et al., 2012). Briefly, the cilium coordinates at rest and under flow were normalized by the length of the cilium and parameterized as a function of the position along the cilium. The observed cilium profile is fit to the deflection predicted by the model using the method of least squares and varying mechanical properties. Specifically, the cilium profile at rest is captured before each bout of flow and used to determine the internal stress within the cilium. The cilium profile with flow at 30 s, 2 min or 10 min is used to extract stiffness at those time points. Protrusion angle, the angle between the cilium and cell membrane, was measured with each bout of flow and change in protrusion angle was used to determine the anchoring torsional stiffness. The instant center of rotation was calculated with each deflection by tracking the motion of two points on the axoneme near the base roughly 1 µm apart (Battle et al., 2015; Spiegelman and Woo, 1987). The pivot point was the resulting center point.

### mRNA expression

RNA was extracted from cell lysate using the Autogen RNA Extraction kit and the Quickgene Mini80 (Autogen). The TaqMan reverse transcription kit (Life Technologies) was used for reverse transcription. Samples were analyzed in triplicate by relative quantitative real-time RT-PCR and expression was normalized to that of housekeeping gene *GAPDH*. Relative quantification of expression levels was determined using the standard curve method with the following primer-probe pairs: *HDAC6* (Mm01341125_m1), *COX-2* (Mm00478374_m1), and *GAPDH* (4352339E).

### Immunocytochemistry

Cells were fixed in 10% formalin and permeabilized with 0.1% Triton-X. Cells were then incubated in primary antibody solution, anti-acetylated α-tubulin (Abcam, 6-11B-1, 1:1000), and the secondary antibody solution, anti-mouse Alexa Fluor 568 (Life Technologies, 1:200). Cells were imaged on a laser scanning confocal microscope (Leica SP5; Leica Microsystems) with a 63× oil objective (1.4 NA). Maximum-intensity *z*-projections were generated with the Leica software.

### Western blots

Cells were lysed in radioimmunoprecipitation (RIPA) buffer (Thermo Scientific) and protein content was measured by bicinchoninic acid assay. Protein was separated by electrophoresis in 4-12% Bis-Tris polyacrylamide gels (NuPage, Life Technologies) and transferred to polyvinyl difluoride membranes. Membranes were probed for acetylated α-tubulin (6-11B-1, 1:1000; Abcam) and actin (AC-40, 1:2000; Abcam). The bound primary antibodies were detected by chemiluminescence with HRP-conjugated secondary antibodies (1:10,000; Millipore).

### Statistical analysis

All data are presented as mean±s.e.m. and analyzed with GraphPad Prism (GraphPad Software). A one-way repeated measures ANOVA was used to assess the effects of duration of flow exposure on cilium stiffness with a Dunn's post hoc test for multiple comparisons. A two-way ANOVA was used to assess effects of siRNA-mediated knockdown and flow on mRNA expression followed by Sidak's multiple comparisons test. Statistical significance was considered at *P*<0.05.
